# Diagnosis and treatment depression in schizophrenia

**DOI:** 10.17650/2712-7672-2020-1-2-29-42

**Published:** 2020-12-04

**Authors:** Sergey N. Mosolov

**Affiliations:** Moscow Research Institute of Psychiatry – a branch of the V. Serbsky Federal Medical Research Centre of Psychiatry and Narcology of the Ministry of Health of the Russian Federation; Russian Medical Academy of Continuous Professional Education of the Ministry of Public Health of Russian Federation

**Keywords:** depression, schizophrenia, therapy, antipsychotics, antidepressants, evidence-based therapeutic algorithm, депрессия, шизофрения, терапия, антипсихотики, антидепрессанты, терапевтический алгоритм на основе доказательных данных

## Abstract

Depression is the third most common illness among patients with schizophrenia which negatively affects the course of the disease and significantly contributes to the mortality rate, due to increased suicide. Depression, along with negative symptoms and cognitive deficits, is one of the main factors that significantly decreases the quality of life and the disease prognosis in patients with schizophrenia. In addition, depression increases the frequency of exacerbations and readmissions, decreases the quality and duration of remissions and is associated with more frequent substance abuse and an increased economic burden. Data on the prevalence of depression among patients with schizophrenia are contradictory and are associated with a low detection rate of depression in such patients, a lack of clear diagnostic criteria and difficulties in differentiation between extrapyramidal and negative symptoms. The average prevalence of depression that meets the diagnostic criteria of major depressive episodes in patients with schizophrenia is 25% at a specific point, and 60% over the course of a lifetime; the frequency of subsyndromal depression is much higher. It is essential to distinguish between primary (axial syndrome) and secondary depressive symptoms (extrapyramidal symptoms, psychogenic or nosogenic reactions, social factors, etc.) to determine treatment strategies.

The published data relating to randomized clinical trials for the development of evidence-based guidelines are limited. Current recommendations are based mainly on the results of small-scale trials and reviews. Certain atypical antipsychotics (quetiapine, lurasidone, amisulpride, aripiprazole, olanzapine, clozapine) are superior to typical antipsychotics in the reduction of depressive symptoms. Clozapine is effective in the management of patients at risk from suicide. The additional prescription of antidepressants, transcranial magnetic stimulation and electroconvulsive therapy are not always effective and are only possible following the management of acute psychosis in cases when antipsychotic monotherapy proved to be ineffective.

Alongside negative symptoms and cognitive impairment, depression is one of the most significant deconditioning factors among patients with schizophrenia, which significantly reduces the quality of life and the disease prognosis as a whole [1-3]. In addition, depression increases the frequency of exacerbations and rehospitalizations, and decreases the quality and duration of remissions; it is associated with more frequent substance abuse, an increased economic burden with regard to the disease and is also the main cause of suicide [4-8]. The risk of suicide among people suffering from schizophrenia is 20 times higher than among the general population; around 50% of patients with schizophrenia attempt suicide and around 10% die from suicide [9]. Data relating to the prevalence of depression among the population of patients with schizophrenia are rather contradictory, due to the low detectability of depression in this category of patients, the lack of clear diagnostic criteria and the difficulties in clinical differentiation between extrapyramidal and negative symptoms. In various studies, the reported data on the prevalence of depression among patients with schizophrenia vary considerably from 25 to 70%, depending on the methodological approaches used [10-12]. On average, the prevalence of depression in schizophrenia at one particular moment is 25% and at lifetime is 54% [13]. Up to 60% of patients with a verified diagnosis of schizophrenia have at least one episode of major depression [14]; 40-50% of both inpatients and outpatients have mild or moderate depressive episodes. Depression can develop at any stage of schizophrenia: depression was observed in the premorbid period before the onset of psychotic symptoms in 50% of patients, in 33% of patients during the first episode, in 38% of patients during psychotic episodes and in 27% of patients in remission [15].

From a historical aspect, it should be noted that the founder of the concept of schizophrenia, E. Bleuler, identified a whole layer of affective states within this disease, including "schizophrenic melancholy", considering it a manifestation of an endogenous process [16]. Moreover, it could be both an independent syndrome and an optional syndrome, developing within a psychotic episode. In addition, Bleuler did not exclude a psychological mechanism of depression as a reaction to psychotic experience, which is often encountered during the first episodes of the disease. Almost all clinicians who have studied depression among patients with schizophrenia indicate that the schizophrenic process makes an impact on the phenomenological manifestations of depression. Bleuler also described the so-called schizophrenic "tension", incompleteness, rigidity, superficiality and pretentiousness of the hypothymic manifestations, associated, in particular, with a limited emotional resonance and an inability of such patients to give vivid, affective responses. A.V. Snezhnevsky also noted the absence of a differentiated feeling of anguish, effacement and incompleteness of affective and vital manifestations among these patients [17]. Indeed, vivid, vital and autonomic symptoms with typical daily fluctuations of mood, are observed less often in such patients; on the contrary, apathy, anergy, mental anaesthesia, dysphoria, gloominess, irritability, grumbling and other atypical manifestations of depression are more common [18,19].

In DSM-III the possibility of independent (comorbid) diagnosis of overt depressive syndrome was determined for the first time within the framework of schizophrenia, in accordance with the criteria of a major depressive episode, with the development of post-schizophrenic depression being diagnosed in remission. This was caused by a number of epidemiological studies in the United States, which showed that syndromal depression among patients with schizophrenia occurs 29 times more often than among the general population [20], and in 59% of patients with schizophrenia, depression meets the criteria for a major depressive episode [21]. Subsyndromal or minor depression that does not meet the criteria of the diagnostic threshold, occurs much more often among 80% of patients with schizophrenia [21]. Meanwhile, subsyndromal depressive symptoms, like major depression, are associated with social and financial problems, a poor quality of life, an increased volume of medical care, a general worsening of symptoms, demoralization, frequent recurrence and an increased suicide risk [13,22,23]. Thus, M. Birchwood et al. [24] prospectively monitored the condition of 105 patients, diagnosed with schizophrenia according to ICD-10, after psychotic episode and on at least five subsequent occasions within 12 months: depression was identified among 70% of patients in an acute psychotic state and reduced simultaneously with a reduction in psychotic symptoms; 36% of patients developed post-schizophrenic depression without the exacerbation of psychotic symptoms and more than half of patients had suicidal thoughts. In accordance with the ICD-10 diagnostic criteria for post-schizophrenic depression [25], in relation to the reduction of psychotic symptoms and the meeting of formal criteria for a depressive episode, such patients may exhibit certain residual symptoms of schizophrenia, primarily negative symptoms.

In accordance with the new dimensional classification paradigm of schizophrenia, depression within the five-factor model is one of the independent domains (dimension) of schizophrenia symptoms, which is less prevalent only than psychotic (positive) and negative symptoms [26]. The modern conceptualization of depression among patients with schizophrenia describes it as one of the key components of schizophrenia [13,27], with some input of secondary psychological reaction to psychosis and/or psychosocial stress [22,28], as well as, to a lesser extent, of neuroleptic side effects (in up to 15% of cases) [29].

Clinically, depression in schizophrenia can be divided into two main categories: related and not related to the psychotic episode. In the first case, depressive symptoms are immediately present in the structure of the psychotic episode and are usually reduced along with psychosis. This example is the most typical and occurs among around 50% of all patients with schizophrenia with depression. Phenomenologically, such depression is usually characterized by apathy, anergy, anhedonia, phenomena of depressive depersonalization and feelings of guilt, although patients often blame others rather than themselves. According to G.E. Mazo, the presence of anergic depression results in a continuous course of the disease and a less favourable prognosis [6]. In certain patients, depression, at the beginning of the psychotic episode, which is usually masked by significant hallucinatory-delusional symptoms, is apparent after the reduction of psychosis as a result of effective, antipsychotic therapy, therefore, a kind of "stratification of the syndrome" and "filtering out" of depression occurs [30] (the so-called "revealed depression") [31].

Depression can also be caused by pharmacogenic factors and resulting from a antipsychotic therapy complications (the so-called 'neuroleptic depression'). Long-term dopamine receptor blockade can lead to the development of anhedonia and, possibly, depression [32]. The data concerning the relationship between neuroleptic medication and the onset of depression are very contradictory, and these observations primarily involved the use of the first generation of antipsychotic drugs - conventional neuroleptics [33,34]. A typical manifestation of neuroleptic depression, along with psychomotor retardation and anhedonia, is the presence of akinetic-rigid symptom complex and other phenomena of neuroleptic pseudoparkinsonism. The addition of akathisia in such patients can cause a temporary change in the modality of the hypothymic affect, with the development of dysphoria and suicidal behavior [35-37]. However, in the literature there are descriptions of pharmacogenic depression without clinically pronounced extrapyramidal symptoms. These include, e.g., "akinetic depression" [38]. In this case, the authors consider akinesia as a new extrapyramidal symptom that is not part of the structure of parkinsonism and is mainly associated with the blockade of dopaminergic neurotransmission at the cortical level. Therefore, previously, we classified these peculiar states, which respond poorly to any thymoanaleptic therapy and are associated with impaired dopamine metabolism by neuroleptic agents, as dopamine-dependent depression [39,40]. Another clinical example of pharmacogenic depression, occurring without clear extrapyramidal symptoms, are conditions that are phenomenologically similar to negative symptoms: apathy, anhedonia, poverty of speech, decreased emotional expressiveness, which, however, respond to antidepressant therapy and are reduced along with a termination of the psychotic episode [41].

Finally, reactive moments play an important role in the development of depression associated with a psychotic episode. Schizophrenic psychosis is a severe psychological burden for patients; therefore, it is not surprising that they often develop reactive states that can be characterized as nosogeny and adjustment disorders. The reasons for this are stigma, the emotional experience of one's own failure, as well as social maladjustment. Certain patients may show symptoms resembling the so-called demoralization or frustration syndrome [42]. It is not always easy to differentiate this syndrome from depression in schizophrenia. It is characterized by feelings of hopelessness and helplessness, combined with self-doubt and feelings of failure. Of course, the most cases of depression in schizophrenia cannot be explained by reactive mechanisms. If we assume the opposite, there would be a direct relationship between the severity of depression and the degree of restoration of a critical attitude to the disease, i.e., depressive symptoms should have occurred more frequently as the psychotic symptoms were reduced by treatment. However, in practice, the opposite has been observed - the symptoms of depression often disappear after the elimination of positive symptoms [43]. Therefore, in the first example, it is important to trace the dynamics of depressive symptoms during psychotic episode and to find out its genesis, its provoking factors, as well as the connection with other psychopathological symptoms.

The second example concerns the development of depression not directly associated with an acute psychotic episode and separated from it by a certain period of time. In these cases, depression should be initially differentiated from primary negative symptoms since depression can be overlaid on these symptoms [44].

A series of studies have shown that negative symptoms and depression have a number of common clinical manifestations that can complicate differential diagnosis [45-47]. Decreased interest, motivation and emotional expression, anhedonia, anergy and psychomotor retardation, as well as cognitive impairment, are all overlapping features of these condition [48]. Nevertheless, there are certain symptoms that make it possible to differentiate between depression and negative syndrome [49-51]. In contrast to affective flattening and abulic negative symptoms, depression is characterized primarily by a distinctly depressed or melancholic mood and specific cognitive impairments, such as depressive ruminations, feelings of helplessness, ideas of guilt and low value of life, which sooner or later lead the patient to suicidal ideas and intentions. In addition, for a more accurate diagnosis, it is necessary to pay attention to the onset of depressive symptoms, their progression and their prevalence in relation to the use of certain drugs [44]. According to a recent study with a multivariate analysis of symptoms [52], hypothymic affect, as well as pessimistic and suicidal thoughts are significantly more common in depression, and in negative symptoms, such as poverty of speech (alogia), flattening of affect and social isolation; other symptoms intersect and cannot be reliable indicators for differential diagnosis. The Calgary Depression Scale for Schizophrenia (CDSS), developed specifically for this purpose, helps significantly to distinguish between depressive and negative symptoms [53]. CDSS surpassed the Hamilton Depression Scale (HAM-D), the PANSS Depression Factor (PANSS-D) and the Beck Depression Inventory (BDI) in terms of its sensitivity and specificity [54].

In recent years, close attention has been paid to depression in schizophrenia as an axial independent syndrome, in particular, outside the stage of psychotic exacerbation. The terms post-psychotic depression, post-schizophrenic depression and secondary depression have been used most frequently to describe these manifestations. Of course, the variety of terms and their interpretations does not add clarity to the understanding of this issue. The diagnostic criteria for post-schizophrenic (ICD-10) or post-psychotic depression (DSM-IV, DSM-V) in modern classifications do not directly link the development of depression with the termination of the psychotic episode and the presence of a stressful psychological reaction to schizophrenic psychosis. Postpsychotic depression is a complex psychopathological formation, and as it develops, residual positive, negative and affective symptoms, as well as reactive personality and pharmacogenic factors, become apparent.

Another fact confirming that depression is an axial syndrome in schizophrenia, is the frequent onset of depression before appearing of psychotic symptoms in the form of a prodrome [13,55]. A.V. Snezhnevsky attributed affective fluctuations to the so-called outpost or 'forpost' symptoms and noted their occurrence even at the premanifest stage of schizophrenia [17]. According to various studies, depending on the severity, depressive symptoms before the first psychotic episode are observed in 20-60% of patients and are an important sign of an impending psychosis manifestation [43,56].

Finally, depressive episodes that meet the criteria for major depression in schizophrenia can develop as an independent syndrome, regardless of psychotic symptoms, then it becomes more common to speak of comorbid depression [57,58]. In fact, comorbid depression in schizophrenia alone, is the purest primary depressive syndrome; when other symptoms occur, secondary mechanisms may play a significant role (according to certain data, up to 80%), including positive (hallucinatory-delusional) and negative symptoms, as well as reactive-personality and pharmacogenic (pseudoparkinsonism and depressogenic effect) factors. For example, depression often develops within the framework of chronic extrapyramidal neuroleptic syndrome, in particular, with tardive dyskinesia and the phenomenon of dopamine hypersensitivity [59].

Another approach that explains the formation of depressive symptoms in schizophrenia is the concept discussed by S.G. Zhislin and G.Ya. Avrutsky relating to pharmacogenic pathomorphosis [30,60]. Long-term exposure to antipsychotics is accompanied by the transition of the course of schizophrenia to the level of affective disorders, with an increase in the phase and circularity factors during the course of the disease.

Specific epidemiological studies carried out in our clinic in the 1960s and 1970s showed an increase in depression among patients with schizophrenia just after spreading of neuroleptic treatment widely. Therefore, in episodic forms of schizophrenia, there was a tendency for prolongation of psychotic episodes, as a result of which, instead of the completion of psychosis, inapparent residual, usually sub-depressive syndromes or so- called neuroleptic depression appeared. Therefore, the development of a number of different types of postpsychotic depressions can be explained in terms of drug pathomorphosis of schizophrenia course and clinical picture [10].

The development of psychopharmacotherapy and other methods of treatment increases the importance of correctly diagnosing depression at an early stages of the disease. The therapeutic goal is to significantly reduce the excess morbidity and mortality associated with depression. An additional objective is to prevent suicide, from which 5 - 15% of patients with schizophrenia die [9,61,62]. The following clinical manifestations correlate with suicide in schizophrenia: depressive symptoms, dependence on psychoactive substances, the severity of psychotic symptoms and cognitive impairment, early stages of the disease, insomnia, agitation and restlessness, as well as a history of depressive episodes and/or suicidal activity [23,63]. According to the recent data, clozapine is the first choice treatment for patients at a high risk of suicide [64,65]. It is believed that the unique anti-suicidal effect of clozapine (many other atypical antipsychotics were ineffective in this respect) is associated less with its antidepressant effect and more with one's own ability to suppress suicidal ideas, which correlates with its specific neurochemical profile, in particular, with antagonism to 5-HT2 and D4 receptors [66].

Pragmatic approaches to the treatment of depression in schizophrenia include the use of various antipsychotic agents or their combination with antidepressant drugs. Although there are several studies and observations in the literature relating to the ability of certain first-generation antipsychotics (FGAs) to reduce depressive symptoms (e.g., with the use of small doses of sulpiride, thioridazine and flupentixol) [67-70], most conventional antipsychotics in therapeutic doses increase the manifestations of depression, primarily due to extrapyramidal side effects and hypersedation [71]. Often, dysphoria also occurs with the development of akathisia or more delineated neuroleptic depression. Therefore, lowering the dose of the antipsychotic agent as a first step can decrease the severity of depressive symptoms [12,72]. Prescribing anticholinergic drugs to correct extrapyramidal symptoms also reduces the severity of depression [46,73].

Pharmacoepidemiological studies show that clinicians usually solve the problem by adding antidepressants, which are prescribed in around 40% of patients both in hospital or outpatient clinics [15,74]. In most cases, selective serotonin reuptake inhibitors (SSRIs) are prescribed, although there are more research data available in relation to tricyclic antidepressants [75,76]. Approximately 30% of physicians prefer combination therapy with atypical antipsychotics and SSRIs. A pharmacoepidemiological study evaluating the frequency of use of antidepressants to treat schizophrenia in Moscow, found that 30-40% of physicians in the dispensary and 70-80% of physicians in the hospital prescribe antidepressants; in 80% of cases these were tricyclic antidepressants, primarily amitriptyline, and SSRIs were used in 14% of patients [77]. However, only 30% of inpatients received adequate therapeutic doses of amitriptyline (> 150 mg/day) and 15-20% of outpatients.

Depressive symptoms during an exacerbation of schizophrenia should not necessarily lead to the prescription of antidepressants, as they are traditionally believed to cause an increase in psychotic symptoms. However, new studies show that the risk of psychosis induction, resulting from antidepressant use is low [78]. There have been very few randomized, double-blind, placebo-controlled clinical trials (RCTs) evaluating the effectiveness and tolerability of combination therapy in the treatment of depression among patients with schizophrenia. One meta-analysis which involved evaluating the effectiveness of tricyclic antidepressants, showed an improvement in only five out of 11 RCTs [76]. In general, according to this metaanalysis, the prescription of a tricyclic antidepressant in combination with an antipsychotic after the relief of acute psychotic symptoms, is associated with a minimal risk of exacerbating positive symptoms, but increases the risk of anticholinergic side effects, due to the pharmacokinetic drug interactions. Several studies have shown that imipramine is the most effective tricyclic antidepressant in the treatment of depression among patients with schizophrenia, possibly due to its distinct psychostimulatory properties [75,79].

Clinical studies of SSRIs have generally confirmed their effect on depressive symptoms in schizophrenia. Sertraline is the only SSRI that has been shown to be effective for the management of depression in 26 stable patients with schizophrenia: the reduction in the mean Hamilton score in the sertraline group was 31% versus 8.6% in the placebo group [80]. However, another RCT, conducted in 48 patients meeting the DSM-IV criteria for schizophrenia in remission and for major depressive episode found a significant placebo effect, which did not prove the effectiveness of sertraline [75,81]. An earlier limited RCT (40 patients) comparing sertraline and imipramine for post-psychotic depression found that they were comparable in efficacy, but sertraline had a faster onset of effect and better tolerance [82]. In general, SSRIs are considered to be effective in treating depression in schizophrenia [72,74]. Considering their relative safety compared to tricyclic antidepressants, they seem to be the drugs of choice. However, it is necessary to bear in mind possible drug interactions with antipsychotics, due to the inhibitory effect of certain SSRIs on the activity of cytochrome P450 [83-85].

There are small positive open-label add-on studies of the selective serotonin-norepinephrine reuptake inhibitors, venlafaxine and duloxetine, to the antipsychotics in resistant post-psychotic depression [86,87], trazodone [88] and dopamine-stimulating drugs [39,40], the use of which, however, is associated with the risk of exacerbation of psychosis, as well as several RCTs of bupropion [89] and a limited RCT of mirtazapine [90].

In the latest meta-analysis evaluating the effectiveness of antidepressants among patients with schizophrenia undertaking antipsychotic therapy, the entire group of SSRIs did not find significant advantages over placebo in the degree of reduction of depressive symptoms in 42 RCTs (1849 patients), however, trazodone, duloxetine, sertraline and amitriptyline were the most effective [91]. At the same time, in more severe depressive episodes (7 RCTs, 422 patients), the entire SSRI group was significantly more effective than the placebo group [91].

In general, most current clinical guidelines do not consider antidepressant drugs as the treatment of choice when treating depression among patients with schizophrenia. Depressive symptoms during acute psychosis are often reduced in parallel with psychotic symptoms, so it makes sense to wait for the antipsychotic effect and not to prescribe an antidepressant too quickly.

The use of antidepressants is only recommended for the treatment of depression in patients with stable chronic schizophrenia, i.e., with persistent depressive symptoms that arise outside of psychosis, while the prescription of antidepressants in an acute psychosis is considered inappropriate [92-96]. The most of the recommendations for prescribing antidepressants as an adjuvant therapy for schizophrenia, have limited evidence of efficacy. The use of antidepressants is recommended in the following cases: 1) when symptoms correspond to a major depressive disorder (symptoms are severe and clinically significant); 2) when symptoms cause stress or affect functioning. Certain methodologically more accurate guidelines highlight the lack of data on the use of novel antidepressants alone or in combination with second generation antipsychotics (SGAs) for the treatment of depression in schizophrenia [72,96]. However, in post-psychotic depression, according to ICD-10 criteria, antidepressant prescription should be discussed on the basis of its clinical appropriateness for the individual patient.

The most interesting and promising approach in the therapy of depression in schizophrenia is associated with the emergence of SGAs, which, due to their multimodal neurochemical action, were found to have an antidepressant effect [58,74]. Unfortunately, in most antipsychotic studies the assessment of depressive symptoms was not the main task, the degree of their severity was not indicated in the inclusion criteria and the reduction of depression was not considered as an efficacy criterion.

A new meta-analysis of the comparative efficacy and tolerability of 32 antipsychotic agents, covering nearly 90 RCTs with 20,000 patients [97] showed that most antipsychotics were significantly superior to placebo in terms of the reduction of depressive symptoms, according the PANSS scale. Obviously, we are referring to depressive symptoms within the structure of an acute psychotic episode, which are reduced along with positive symptoms and are closely related to it. Sulpiride, clozapine, amisulpride, olanzapine, aripiprazole, cariprazine and paliperidone had the most significant effect (in descending order). The question of the superiority of SGAs over FGAs in terms of the reduction of depressive symptoms in schizophrenia remains controversial. There are surprisingly few quality RCTs investigating the effectiveness of SGAs in the treatment of depressive episodes among patients with schizophrenia [98]. In one of them, e.g., quetiapine showed no significant differences compared with haloperidol [99]. A number of studies have also failed to establish differences between haloperidol and SGAs (in particular, risperidone) in the reduction of depressive symptoms [100,101]. At the same time, a meta-analysis, based on 50 RCTs, demonstrated the significant superiority of a number of SGAs (amisulpride, aripiprazole, clozapine, olanzapine, quetiapine) over FGAs in terms of the reduction of depressive symptoms on the PANSS scale in acute episodes of schizophrenia [102]. In this meta-analysis, there were no new SGAs that appeared recently on the market. Lurasidone and cariprazine also have thymoanaleptic properties; in a pooled analysis of four RCTs, lurasidone was superior to placebo in terms of its effect on depressive symptoms in the treatment of exacerbations of schizophrenia within six weeks [103]. At the same time, the reduction in depression only slightly correlated with the decrease in the PANSS scores, which indicates the independent antidepressant effect of lurasidone, which is not associated with its antipsychotic effect. We performed a specific analysis of the effect of lurasidone on symptoms according to the PANSS scale within a five-factor model of schizophrenia who participated in short-term RCTs of the drug in Russia and Ukraine, similar to previous trials with other SGAs [104]. It transpired that the symptoms of depression and anxiety in the local sample were reduced beginning from Week 1 of the therapy, with an even greater effect [105] than in the global world sampling [106].

There have been practically no direct comparative RCTs of FGAs and SGAs in schizophrenia, evaluating their efficacy against depressive symptoms. In a large- scale independent 18-month CATIE study among patients with schizophrenia with severe symptoms of depression, quetiapine was significantly more effective than risperidone in terms of the reduction of depressive symptoms [107]. There is evidence of the superiority of quetiapine over risperidone in terms of the effect on depressive symptoms in schizophrenia [108]. In a comparative RCT of cariprazine and risperidone, evaluating the reduction of primary negative symptoms, no differences were found in relation to the effect of the drugs on depressive symptoms [109]. In one open RCT, it was found that replacing risperidone therapy with amisulpride therapy leads to a decrease in the severity of depression among patients with schizophrenia, compared with patients who continued administering risperidone [110]. When clozapine was compared with other antipsychotics in combination with an antidepressant or placebo, patients treated with clozapine were less depressed [98].

Certain of the aforementioned FGAs and SGAs (in particular, in the case of quetiapine, aripiprazole, amisulpride and sulpiride) have been shown to be effective in the treatment of a depressive episode in recurrent depressive disorder [111], and in the case of quetiapine and lurasidone, in bipolar depression [112,113]. Although it is difficult to extrapolate these findings to patients with schizophrenia, drugs with a high affinity to D2 receptors appear to be less effective in the treatment of comorbid depression (or may even increase symptoms when taken in high doses), while a blockade of 5-HT2 receptors and a partial agonism to D2/8 receptors are associated with a more pronounced thymoanaleptic effect [114].

In addition, in clinical practice involving patients with schizophrenia, it can be difficult to differentiate secondary negative symptoms associated with depression, therefore, in the absence of a response to the adequate antipsychotic and antidepressant therapy in such patients, the strategy recommended for the treatment of persistent negative symptoms can be considered [115].

Among the non-pharmacological therapeutic methods for pharmaco-resistant depression in schizophrenia, the most studied are electroconvulsive therapy (ECT) and high-frequency cyclic transcranial magnetic stimulation (rTMS). The American Psychiatric Association (APA) usually recommends ECT for the treatment of patients with schizophrenia with comorbid depression and/or suicidal ideation in situations where emergency therapeutic intervention is required. At the same time, data from systematic reviews that analysed 31 studies of ECT use in schizophrenia were contradictory and generally confirmed its rapid effect on depressive symptoms, but not on suicidal behavior [116,117]. TMS has been recommended for the treatment of depressive episodes in recurrent depression [118], but there are insufficient data on its efficacy in the treatment of depressive symptoms in schizophrenia. A systematic Cochrane review of five studies with limited samples, identified a small-scale beneficial effect of rTMS [119]. In our clinic, in an open-label study of 15 Hz rTMS of the left dorsolateral prefrontal cortex, the method has also shown to be effective in the treatment of outlined depression in schizophrenia [120-122].

However, a large multicentre RCT with pseudo-TMS control, which included 157 patients with schizophrenia with a predominance of negative symptoms, did not identify the beneficial effect of 10 Hz rTMS of the left dorsolateral prefrontal cortex on depressive symptoms [123]. Among other non-pharmacological methods of biological therapy in our clinic, the effectiveness of intravenous laser blood irradiation [124,125] and adaptation to periodic normobaric hypoxia [126] in post-psychotic depression resistant to psychopharmacotherapy, was also identified.

In addition to biological therapies, attention should be drawn to the proven effectiveness of exercise, including fitness, in reducing depression among patients with schizophrenia [127], as well as cognitive behavioural therapy [128,129] and psychosocial rehabilitation measures [129].

Thus, a rational approach to the treatment of depression in schizophrenia follows the differential diagnosis and the determination of the contribution of reactive-personality and pharmacogenic factors to its development. If a patient receiving FGA has an episode of depression, the question arises as to how much antipsychotic therapy is responsible for the symptoms similar to depression, both extrapyramidal (akinesia or akathisia) and dysphoria directly induced by neuroleptic agents. This problem can be solved in three ways: 1) reduction of the antipsychotic dose, subject to the time available to do it safely; 2) adding and dose titration of antiparkinsonian (anticholinergic) drug, benzodiazepine or beta-blocker (the latter are effective for akathisia); 3) replacement of FGA with SGA. If a patient already receiving SGA develops an episode of depression, the same approaches apply. Dose reduction and the addition of anticholinergic agents are most advisable when using SGA, which have dose-dependent extrapyramidal side effects (risperidone, amisulpride, ziprasidone, cariprazine). An alternative option is to replace one SGA with another. In patients with schizophrenia havingnopositiveeffectofantiparkinsonian drugs, antidepressants can be prescribed to achieve the desired result. Based on the aforementioned analysis of the literature data and in accordance with the principles of evidence-based medicine, we proposed an algorithm for the treatment of depression in schizophrenia [130,131], the latest version of which is shown in the Figure 1. The algorithm indicates the levels of evidence and strength of recommendations for proposed interventions, as recommended by the World Federation of Societies for Biological Psychiatry (WFSBP) [132].

**Figure 1:**
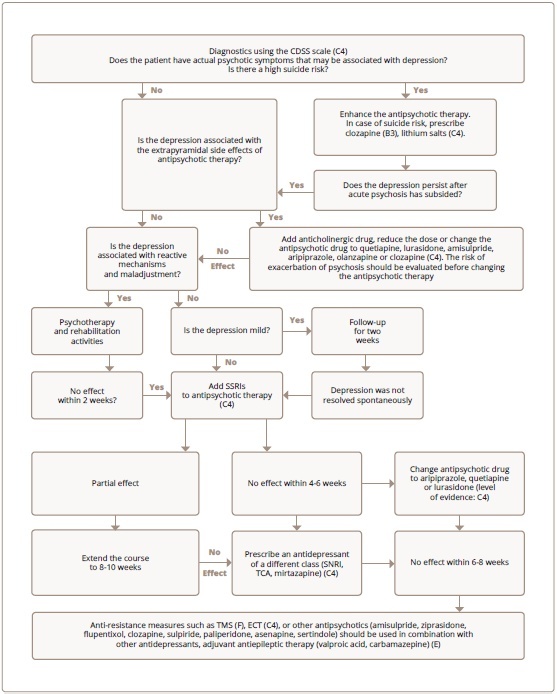
Figure 1. Algorithm for the treatment of depression in schizophrenia with a degree of evidence and recommendation

In conclusion of this review, the following moments, that are important for clinical practice, should be noted. Depression is the third most frequent syndrome of schizophrenia, significantly complicating the prognosis and course of the disease, which considerably contributes to the mortality rate in such patients. It is necessary to distinguish between primary (axial syndrome) and secondary depression (extrapyramidal symptoms, personality reaction, nosogeny, social factors, somatogeny, etc.), which determine the choice of therapeutic strategies. Since comorbid depression has a decisive influence on the increase in mortality among patients with schizophrenia, the clinician is required to ensure a correct diagnosis in a timely manner, prompt intervention in accordance with existing guidelines and close monitoring of the patient's state. There are very few RCT data available in the literature to formulate robust evidence-based recommendations; clinical guidelines are mainly based on literature reviews and are limited by the amount of studies. Certain SGAs (quetiapine, lurasidone, amisulpride, aripiprazole, olanzapine and clozapine) reduce depressive symptoms better than FGAs. If there is a suicide risk, clozapine is preferred. Treatment with the antidepressants, TMS and ECT, is not always effective and is only possible after the relief of acute psychotic symptoms and the ineffectiveness of SGA monotherapy.

Additionally, further well-designed RCTs are needed to develop more evidence-based clinical guidelines.

## Conflict of interest

This work was carried out with a sponsorship from Angelini Pharma Rus LLC.
